# Practical Notes and Formulæ

**Published:** 1886-10-20

**Authors:** 


### PRACTICAL NOTES AND FORMULÆ.

**For Diarrhœa.**—

℞. Pulv. kino .................................. gr. xxx,
Pulv. opii .................................. gr. ij,
Pulv. cinnamomi .................................. gr. viij,
Divide into eight powders.

M. S.—One powder every three hours. This is the "Pulvis Kino Compositus" of the British Pharmacopœa.—*Medical World.*

℞. Pulv. rhei. co .................................. ʒ j,

Sodæ carbonat .................................. gr. xx,

Tinct. opii .................................. m xij,

Aq. menth. pip .................................. ʒ x.

M. S.—For one dose.

℞. Sodæ bicarbonatis .................................. ℈ ij,
Tinct. zingiberis .................................. ss,
Sp. ammon. arom .................................. ʒ j,
Tinct. opii .................................. *m* viij,
Aq. menth .................................. ℥ jss.
M. S.—For one dose.—*Ibid.*

℞. Acid. hydrochloric dil .................................. ʒ j,
Tinct. nucis vomicæ .................................. *m* xlviij,
Aq. menth. pip .................................. ℥ viij.
M. S.—Two tablespoonfuls thrice daily, in nervous diarrhœa.

**Scarlet Fever and Diphtheria.**—Dr. C. R. Illingworth says, in the *Medical Press and Circular*, that the biniodide of mercury is a specific and prophylactic for scarlet fever and diphtheria. He gives it thus:

℞. Sol. hydrag. bichlor .................................. ʒ iij,
Potass. iodid .................................. gr. x,
Ferri am. citrat .................................. gr. xx,
Syrupi .................................. ℥ ss,
Aquam ad .................................. ℥ ij.
Fiat mist. Sig.—One teaspoonful every two hours (for a child of from two to four years).

As soon as all the membranous deposits has disappeared from the parts affected, I give the usual steel and chlorate of potash mixture. As a rule, this occurs in from four to five days, but in severe cases it takes ten.

The only and important exception to this rule of treatment is in those cases where the disease is ushered in with vomiting and purging with scanty rash and collapse. In these, which evidence a rapid liquefaction of the blood by the action of the poison, the iron and chlorate of potash mixture should be given at once in full doses every two hours.—*American Practitioner.*

